# The involvement of *PybZIPa* in light-induced anthocyanin accumulation via the activation of *PyUFGT* through binding to tandem G-boxes in its promoter

**DOI:** 10.1038/s41438-019-0217-4

**Published:** 2019-12-01

**Authors:** Hainan Liu, Jun Su, Yangfan Zhu, Gaifang Yao, Andrew C. Allan, Charles Ampomah-Dwamena, Qun Shu, Kui Lin-Wang, Shaoling Zhang, Jun Wu

**Affiliations:** 1 Centre of Pear Engineering Technology Research, State Key Laboratory of Crop Genetics and Germplasm Enhancement, Nanjing Agricultural University, 210095 Nanjing, China; 2 Institute of Horticulture, Yunnan Academy of Agricultural Sciences, 650205 Kunming, China; 3 The New Zealand Institute for Plant & Food Research Limited, Auckland, New Zealand; 4 School of Biological Sciences, University of Auckland, Auckland, New Zealand

**Keywords:** Light responses, Plant biotechnology

## Abstract

To gain insight into how anthocyanin biosynthesis is controlled by light in fruit, transcriptome and metabolome analyses were performed in the Chinese sand pear cultivar “Mantianhong” (*Pyrus pyrifolia*) after bagging and bag removal. We investigated transcriptional and metabolic changes and gene-metabolite correlation networks. Correlation tests of anthocyanin content and transcriptional changes revealed that 1,530 transcripts were strongly correlated with 15 anthocyanin derivatives (*R*^2^ > 0.9, *P*-value < 0.05), with the top 130 transcripts categorized as being associated with flavonoid metabolism, transcriptional regulation, and light signaling. The connection network revealed a new photosensitive transcription factor, *PybZIPa*, that might play an important role during light-induced anthocyanin accumulation. The overexpression of *PybZIPa* promoted anthocyanin accumulation in pear and strawberry fruit as well as tobacco leaves. Dual luciferase and Y1H assays further verified that PybZIPa directly activated the expression of *PyUFGT* by binding to tandem G-box motifs in the promoter, which was key to differential anthocyanin accumulation in debagged pear skin, and the number of G-box motifs affected the transcriptional activation of *PyUFGT* by *PybZIPa*. The results indicate that the light-induced anthocyanin biosynthesis regulatory mechanism in pear differs from that described in previous reports suggesting that a *bZIP* family member co-regulates anthocyanin biosynthesis with other transcription factors in apple and *Arabidopsis*. It was found that, in response to light, *PybZIPa* promoted anthocyanin biosynthesis by regulating important transcription factors (*PyMYB114*, *PyMYB10*, and *PyBBX22*) as well as structural genes (*PyUFGT*) via binding to G-boxes within promoters. This activation was amplified by the self-binding of *PybZIPa* to activate its own promoter. Overall, we demonstrate the utility of a multiomics integrative approach for discovering new functional genes and pathways underlying light-induced anthocyanin biosynthesis.

## Introduction

Red-skinned pear germplasm resources are relatively rare, yet the red skin trait has high commercial and breeding value. Asian red pear coloration is greatly affected by environmental factors, such as light and temperature. The environmental influence results in unstable coloration, which affects the value of red pears. Red skin in pears is the result of anthocyanin accumulation, as determined by the biosynthesis levels and distribution of anthocyanin. Anthocyanins among the most studied plant compounds, and their metabolic mechanisms have been widely reported. Light is an important environmental factor affecting anthocyanin biosynthesis according to the photoperiod, light intensity and light quality. Light effects on anthocyanin levels have been reported in litchi (*Litchi chinensis*)^1^, grape^2^, waxberry (*Myrica rubra*)^3^, huckleberry (*Vaccinium* spp.)^4^, raspberry (*Rubus idaeus*)^5^, and many other species. In Rosaceae plants such as strawberry^6^, peach^7,8^, pear^9–11^, and apple^12–14^, the effect of light on anthocyanin biosynthesis is significant. Researchers have made progress in exploring the mechanism of the activation of light-induced anthocyanin biosynthesis. In *Arabidopsis*, Constitutively photomorphogenic 1 (COP1) is a “central regulator” of light signal transduction pathways that repress plant light-induced morphogenesis in the dark and interacts with upstream light receptor proteins (CRY1, CRY2, PHYA, PHYB, and UVR8 through its WD40 domain) and downstream target proteins (ubiquitin/26S proteasome system)^15–17^. The ubiquitination of COP1 is important during photomorphogenesis, including anthocyanin accumulation. Among its downstream target proteins/transcription factors, the transcript abundance of LONG HYPOCOTYL 5 (HY5, a bZIP transcription factor) is directly related to photosensitive morphology, UV-B resistance and anthocyanin biosynthesis^18,19^. Recently, it has been shown that HY5 interacts with B-Box proteins (BBXs) to regulate anthocyanin biosynthesis. The relationships between HY5 and BBXs are diverse, and they both transcriptionally regulate each other and form HY5-BBX complexes to modulate activity^20–23^.

Numerous metabolic pathways of plants are influenced by light;^24–26^ however, the regulatory network of light-induced anthocyanin biosynthesis remains to be clarified. The development of advanced technologies in the fields of functional genomics, metabolomics, proteomics, and epigenetics has helped to provide insights into such complex pathways. More recently, multiomics integration analysis has been applied to study plant development^27–29^, environmental responses^30–33^, and other biological processes^34–36^. Specifically, transcript and metabolite datasets have been analyzed and compared, integrated through correlation and cluster analyses and ultimately used to reveal the relationships between genes and metabolites in *Arabidopsis*^30,35,37,38^ and fruit trees such as citrus^29,32,39^, grape^40,41^, kiwifruit^42^, lichi^43^ and fig fruit^44^ trees. Integrated analysis of “omics” datasets (i.e., transcriptome and metabolome) can provide an effective strategy for identifying potential genes regulating light-induced anthocyanin biosynthesis in red Chinese sand pear.

The anthocyanin biosynthesis mechanism of pear has been described, including the genes encoding the biosynthetic enzymes (e.g., *ANS*, *DFR*, *UFGT*) and some regulatory transcription factors (such as MYB-bHLH-WD40, MYB-bHLH-ERF) involved^45,46^. However, fewer studies have shown the differential expression of genes or metabolites during light-affected anthocyanin biosynthesis. Here, we explored the regulatory pathways of light-induced anthocyanin biosynthesis in pear at the transcriptional and metabolic levels. We focused on the differentially accumulated anthocyanin metabolites in fruit and their related regulatory genes between the time of bagging and bag removal. Connection networks were generated based on correlation analyses between anthocyanin metabolites/derivatives and transcripts associated with the anthocyanin metabolic pathway. Our findings provide new information about light-induced anthocyanin biosynthesis and its regulatory mechanisms in pear and highlight the significance of an integrated multiomics approach for understanding plant physiological processes.

## Results

### Anthocyanin accumulates rapidly in pear after bag removal

**Fig. 1:**
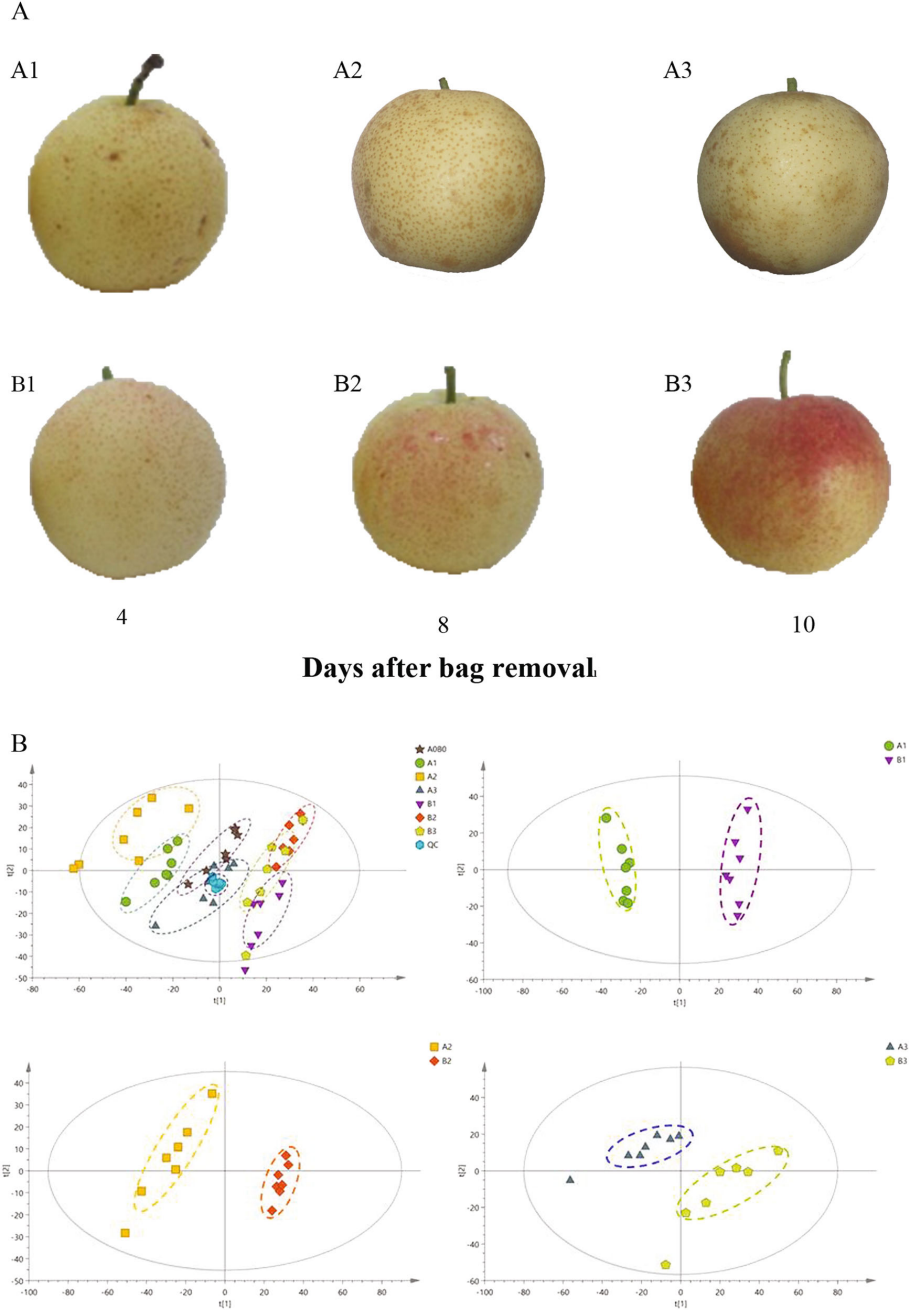
The phenotype of ‘Mantianhong’ fruits and principal component analysis (PCA) scores of peel metabolites. **a** Bagged (A1, A2, A3) and debagged (B1, B2, B3) “Mantianhong” fruit at 4, 8, and 10 days after bag removal (fruits were bagged 35 DAFB). Bagged pear fruits were kept in the paper bags until they were observed and collected. Debagged pear fruits were grown under natural light for 4, 8, and 10 days after bag removal. **b** PCA of peel metabolites from different coloration stages in the bagged and debagged groups.

The skin of bagged “Mantianhong” fruits (fruits were bagged 35 days after full bloom (DAFB)) appeared yellow-white without subsequent debagging and light re-exposure. The fruit after bag removal showed red coloration, and this was more obvious in the fruit skin after light re-exposure in the debagged group (Fig. 1a). Metabolite composition datasets were subjected to PCA comparisons. PCA of all metabolites revealed clear separation of the debagged and bagged samples and clear separation of samples from different stages (Fig. 1b). The *R*^*2*^*X* values between B1/A1, B2/A2, and B3/A3 were 0.610, 0.602 and 0.569, respectively, which showed that the model was reliable (*R*^2^*X* > 0.4). The debagged and bagged samples from different stages were well distinguished and well clustered by the model. There was a difference between each group (Fig. 1b), and the samples presented good repeatability, indicating that the model can be used for qualitative and quantitative research. We identified 89, 94, and 71 differentially accumulated metabolites in B1/A1, B2/A2 and B3/A3, respectively. The global metabolite changes during ‘Mantianhong’ fruit coloration were represented in a heatmap (Fig. S1), which clearly differentiated the bagged and debagged groups. These substances included sugars and glycosides, organic acids, amino acids and other substances. The contents of sugars and anthocyanin biosynthesis-related glycosides increased significantly in the debagged treatment. A total of 15 anthocyanin derivatives, including cyanidin (Cy), apigenin (Ap), and some intermediate products (quercetin, kaempferol, catechin, etc.), were found to be differentially accumulated (Table S2). Among these derivatives, Cy was found in all debagged fruit, and Cy derivatives were important pigments in red pear peels after bag removal.

### Anthocyanin biosynthesis and light response-related genes are differentially expressed between bagging and debagging treatment

**Fig. 2:**
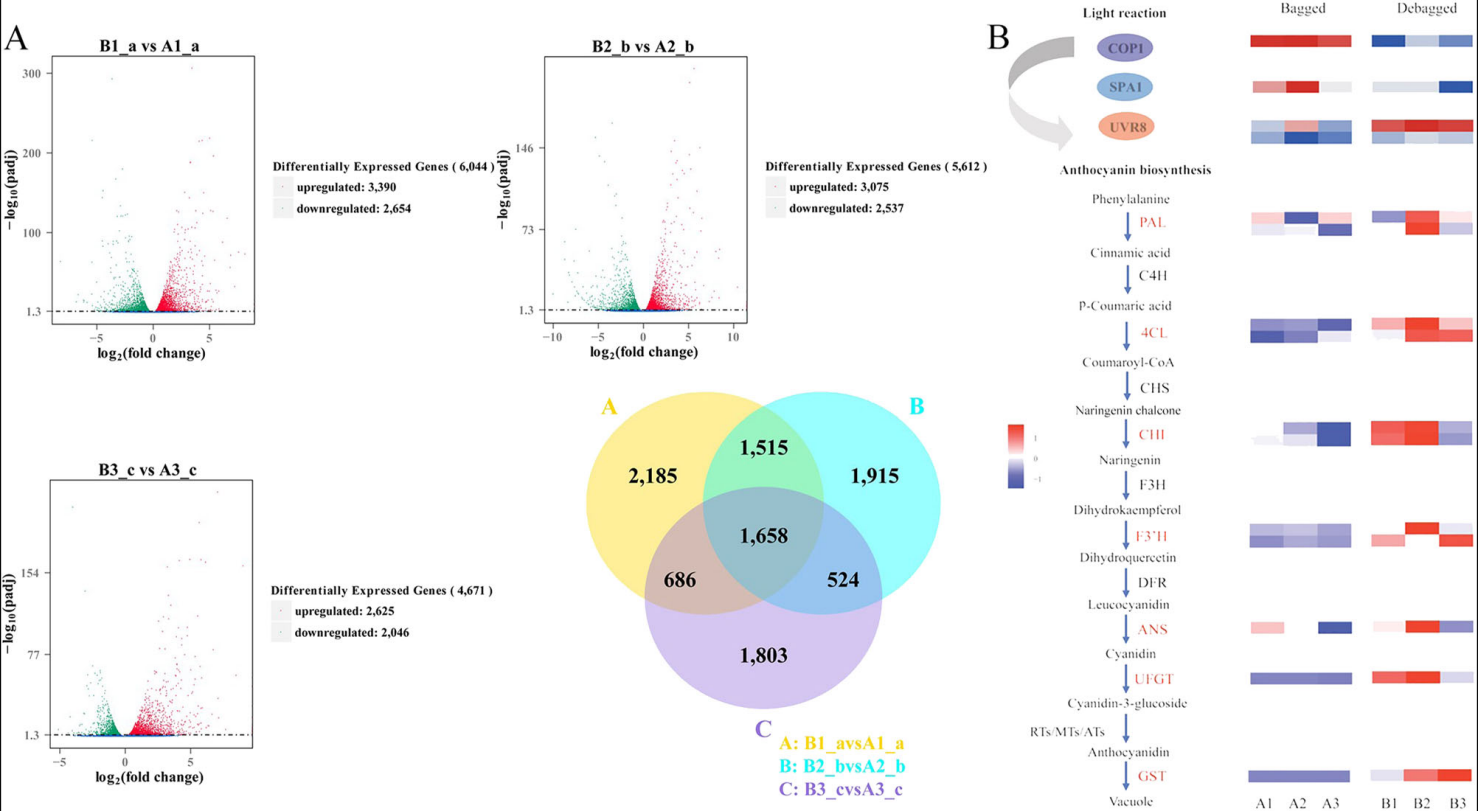
The differentially expressed anthocyanin biosynthesis and light response-related genes. **a** Venn diagram and volcano plot of the DEGs from the transcriptome. **b** Anthocyanin metabolism pathway and structural gene expression in the bagged and debagged groups. COP1 (Constitutively photomorphogenic 1), SPA1 (SUPPRESSOR OF phyA-105), and UVR8 (UV-B resistance 8). Phenylalanine ammonia-lyase (*PAL*), two 4-coumaroyl-CoA ligase (*4CL*), two Chalcone isomerase (*CHI*), two Flavonoid 3′-hydroxylase (*F3*′*H*), one anthocyanidin synthase (*ANS*), one UDP-glucoside: flavonoid glucosyltransferase (*UFGT*), and one glutathione S-transferase (*GST*) gene.

We found that 3,390, 3,075, and 2,625 genes were significantly upregulated, while 2,654, 2,537, and 2,046 genes (new transcripts included) were significantly downregulated in B1/A1, B2/A2, and B3/A3, with 1,658 DEGs being common to all three comparisons (Fig. 2a). Hierarchical clustering analysis of DEGs indicated that the pericarp could be sorted into two different groups according to the bag-removal treatment (Fig. S2). Overall, bag-removal had a significant influence on the global gene expression pattern of “Mantianhong” fruit.

The common DEGs were involved in flavonoid and phenylalanine metabolic pathways and comprised light response-related genes. These genes included 11 anthocyanin biosynthesis genes and several light response center genes (Fig. 2b), two that encoded phenylalanine ammonia-lyase (PAL), two 4-coumaroyl-CoA ligase (4CL), two chalcone isomerase (CHI), two flavonoid 3′-hydroxylase (F3′H), one anthocyanidin synthase (ANS), one UDP-glucoside:flavonoid glucosyltransferase (UFGT), and one glutathione S-transferase (GST). A light response switch gene (*COP1*) and other light-responsive genes (*SPA1* and *UVR8*) were also included.

### Correlation analysis of differentially expressed transcripts and anthocyanin derivatives

**Fig. 3:**
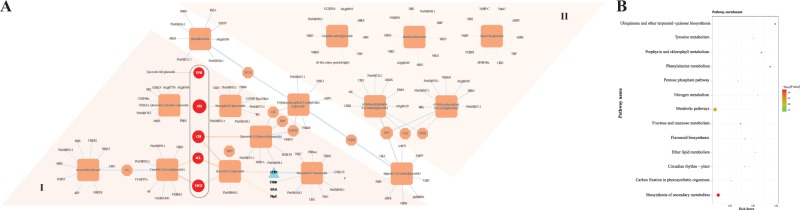
Integrative analysis between anthocyanin-related metabolites and common DEGs. **a** Integrative analysis of the metabolome and transcriptome. **b** KEGG pathway enrichment analysis of metabolism-related genes.

To understand the regulatory network between the bagged and debagged groups, correlation analysis between metabolites and transcripts was carried out. For this analysis, the mean content of the derivatives of anthocyanins and the mean expression levels of common differentially expressed transcripts were used for the correlation tests. In total, 15 anthocyanin derivatives and 1658 common DEGs were subjected to Pearson correlation analysis. A total of 1530 DEGs presented strong correlation coefficient values (Pearson correlation coefficient > 0.9 and *P*-value < 0.05) with 15 metabolites. Among the biosynthesis genes, the gene with the highest correlation coefficient (Pearson *r* = 0.999) between its expression pattern and anthocyanin content was Pbr003128.1, which encodes a F3′H, followed by Pbr003752.1 and Pbr001279.1, which encode a Chalcone isomerase and UFGT, respectively, with correlation coefficients of 0.978 and 0.966. In addition, members of the light response complex (*COP1*, *SPA1*, *PhyE*, and *UVR8*) exhibited expression levels that correlated well with anthocyanin biosynthesis. This further supported the role of light in anthocyanin regulation. Ten transcripts were selected for each anthocyanin derivative (a total of 130 transcripts after removing duplicates) (Table S2) and visualized using Cytoscape (version 3.4.0), and the 15 metabolites were divided into two groups (I–II) (Fig. 3a). The metabolites in group I, containing derivatives of Cy and other intermediate products (four Quercetin, Kaempferol, etc.), were more predominant in the debagged group and presented high correlations with anthocyanin biosynthesis structural genes. The metabolites in group I were also highly correlated with the light response switch gene (*COP1*) and other light-responsive genes (*SPA1*, *PhyE* and *UVR8*). The top 130 transcripts for the 15 anthocyanin derivatives were subjected to KEGG pathway enrichment analysis, showing that phenylalanine metabolism; flavonoid biosynthesis; porphyrin, and chlorophyll metabolism; metabolic pathways; and Fructose and mannose metabolism were differentially enriched (Fig. 3b).

### *PybZIPa* plays an important role in light-induced anthocyanin biosynthesis

**Fig. 4:**
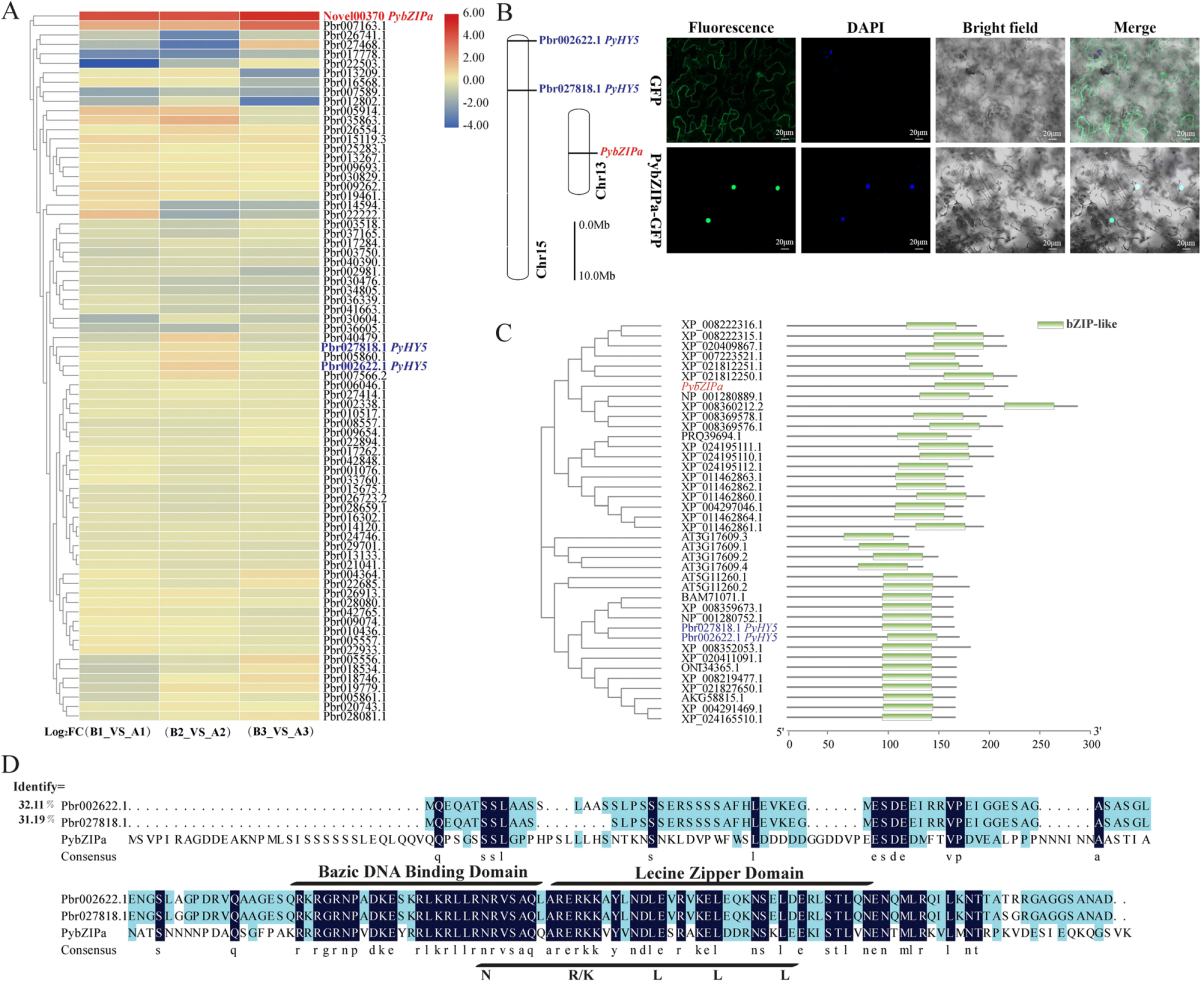
Comparative analysis of *PybZIPa*. **a** Log_2_ fold change (Debagged/Bagged group) of *PybZIP*. **b** Chromosome location and subcellular localization of *PybZIPa*. **c** Phylogenetic relationships and architecture of conserved protein motifs in *HY5* genes from *Arabidopsis thaliana* and other Rosaceae fruits. **d** Sequence alignment between *PybZIPa* and two annotated *PyHY5*.

From the correlation analysis, we identified a highly expressed novel transcript (which encoded a basic leucine zipper domain (bZIP)-type transcription factor and was considered an anthocyanin-associated *bZIP* gene, *PybZIPa*). This gene was highly correlated with Cy in group I, and its expression level in the debagged groups was significantly higher than that in the bagged groups (with a log_2_ fold-change between the debagged and bagged groups of nearly 5). There are 89 *bZIP* genes in pear (according to the family assignment rules from PlantTFDB) and 74 *bZIP* genes were expressed in pear skin after bag removal, with the *PybZIPa* expression level being much higher than those of other *bZIP* homologous genes in the debagged groups (Fig. 4a). *PybZIPa* was located on Chr13, whereas the two *bZIP* genes (Pbr002622.1 and Pbr027818.1) annotated as *PyHY5* (Long Hypocotyl 5) were located on Chr15 (Fig. 4b). To determine the subcellular localization of the PybZIPa protein, *PybZIPa-GFP* and the empty GFP vector (as a control) were transferred to *Nicotiana benthamiana* by *Agrobacterium*-mediated transformation. The green fluorescence signal from *PybZIPa-GFP* in epidermal cells was detected specifically in the nucleus. However, the signal from the empty GFP vector was detected throughout the cell (Fig. 4b). These results indicated that the PybZIPa protein was a nuclear protein.

**Fig. 5:**
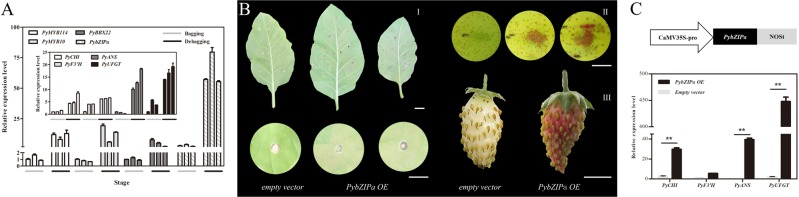
Expression and overexpression of *PybZIPa*. **a** Expression analysis of *PybZIPa* coexpressed structural genes and transcription factors in the bagged and debagged groups (*Tub* was used as a reference gene, the 2^−ΔΔCp^ algorithm was used for the calculation of relative gene expression levels). **b** Overexpression of *PybZIPa* in tobacco, pear and strawberry. **c** Expression analysis of pear tissue transiently expressing *PybZIPa*.

Furthermore, the phylogenetic relationships and architecture of the conserved protein motifs from *Arabidopsis thaliana* and other Rosaceae fruits showed that *PybZIPa* differed from the annotated *PyHY5* in pear (Fig. 4c). The comparison of the nucleic and amino acid sequences of *PybZIPa* and these two *PyHY5* sequences showed only ~32% similarity (Fig. 4d). The comparative analysis of protein properties showed that *PybZIPa* exhibited a different predicted secondary structure, a lower isoelectric point, and more phosphorylation sites (Table S3). qRT-PCR further confirmed that the transcript levels of *PybZIPa* and candidate structural genes were upregulated during anthocyanin biosynthesis and were significantly induced by light re-exposure (Fig. 5a).

The expression patterns of anthocyanin biosynthesis-related transcription factors (*PyMYB10*^10^, *PyMYB114*^46^, and *PyBBX22*, whose homologs are reported to be involved in anthocyanin biosynthesis in apple^23^ and rice^47^) were analyzed (Fig. 5a). *PybZIPa* was coexpressed with the anthocyanin biosynthesis-related transcription factors with correlation coefficients of approximately 0.77–0.93 (average correlation coefficient = 0.83) (Table S4), suggesting possible relationships between *PybZIPa* and *PyMYB10*, *PyMYB114* and *PyBBX22*. To confirm this, we performed a dual-luciferase assay.

The overexpression of *PybZIPa* in pear, strawberry fruit, and tobacco leaves increased pigmentation in these tissues (Fig. 5b). Tobacco leaves showed only a slight increase in anthocyanin accumulation, while more anthocyanins accumulated in the transiently transformed Rosaceae fruits (strawberry and pear fruit). These phenotypes provide evidence that *PybZIPa* plays a role in anthocyanin biosynthesis. Four anthocyanin biosynthesis genes were coexpressed in the bagged and debagged pear samples, which were analyzed in pear tissue transiently expressing *PybZIPa* (Fig. 5c). *PyUFGT* was the most upregulated under the overexpression of *PybZIPa* (their expression patterns exhibited the strongest correlation (0.93, *P*-value < 0.05)), while there was no significant increase in *PyF3*′*H* expression, which indicated that there was no direct regulatory relationship between *PybZIPa* and *PyF3*′*H*. Therefore, we inferred that *PyUFGT* was most likely of these genes to be regulated by *PybZIPa*.

**Fig. 6:**
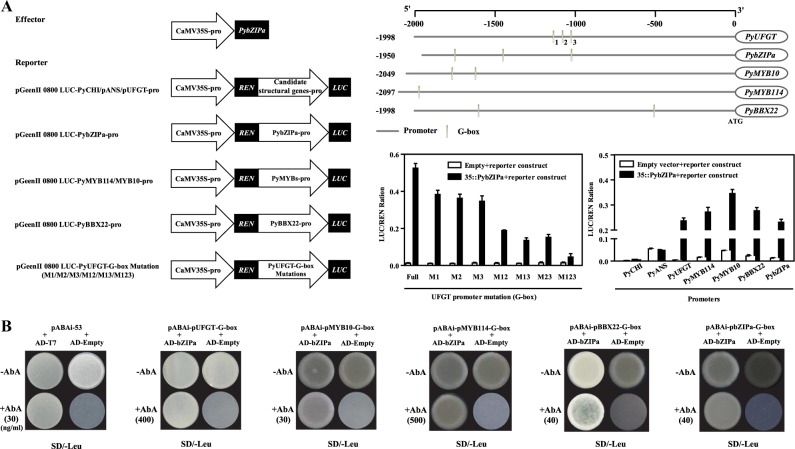
*PybZIPa* upregulates *PyUFGT* and anthocyanin biosynthesis-related transcription factors by binding to G-box elements in their promoters. **a** Vector construction for effectors and reporters, cis-acting regulatory element analysis of promoters, and transcriptional activation activity detection. **b** Yeast one-hybrid analysis. Notes: The G-box motifs in pro-UFGT are numbered 1, 2, and 3 according to their location, and M1/M2/M3/M12/M13/M23/M123 represent the corresponding G-box that was mutated.

To understand the transcriptional activation of *PyUFGT* by *PybZIPa*, the *PyUFGT* promoter and other coexpressed genes were analyzed by using the PlantCARE database. The results indicated that the *PyUFGT* promoter contained tandem repeats of G-boxes (Fig. 6a). Two and one G-box motif were found in the *PyCHI* and *PyANS* promoters, respectively. Considering that four coexpressed anthocyanin biosynthesis genes were selected for expression analysis in the bagged and debagged pear samples and the presence of G-motifs in the *UFGT* promoter region, which may be target motifs of *PybZIPa*, at least *PyUFGT* potentially represents a strong candidate for the *PybZIPa* target (correlation coefficient = 0.93) (Table S4); therefore, we performed that transient LUC assay to confirm our speculation. According to reported regulatory elements, we speculated that *PybZIPa* binds to the G-box elements in the *PyUFGT* promoter. Approximately 2 Kb of the *PyUFGT* promoter was cloned into pGreen 0800-LUC to test for transcriptional activation by *PybZIPa*. Dual luciferase assay results showed that *PybZIPa* increased the transcriptional activity of the *PyUFGT* promoter (Fig. 6a). To verify the effects of tandem G-box motifs on *PybZIPa* binding, we introduced single and multiple G-box motif mutations in the *PyUFGT* promoter. Dual luciferase assay results showed that the transcriptional activation of the *PyUFGT* gene by *PybZIPa* depends on the number of G-box motifs in the *PyUFGT* promoter. When either one of the tandem G-box motifs was mutated, the transcriptional activation activity of *PybZIPa* decreased by ~30%. The activation activity of *PybZIPa* decreased with a decreasing number of G-box motifs (Fig. 6a). At the same time, we tested the transcriptional activity of *PybZIPa* at the *PyCHI and PyANS* promoters, but their transcriptional activity did not show a significant increase even when the G-box motif was present. Compared with the *PyCHI* and *PyANS* promoters, G-box motifs are repetitively distributed in the *PyUFGT* promoter, which is important for promoter activation by *PybZIPa*, as shown above. The lower number of G-boxes may lead to a weak activation effect of *PybZIPa* on the *PyCHI* and *PyANS* promoters. On the other hand, the recruitment of coregulators or coactivators may be required^20^, or epigenetic modification of *PyCHI* and *PyANS* may be involved in their regulation.

Consistent with the coexpression results between *PybZIPa* and anthocyanin biosynthesis-related transcription factors (*PyMYB114*, *PyMYB10*, *PyBBX22*), there was at least one G-box motif present in their promoters. *PybZIPa* showed different degrees of activation of these promoters in the dual-luciferase assay (Fig. 6a). In addition, *PybZIPa* can activate its own promoter, and the high expression level of *PybZIPa* is likely due to this positive autoregulation.

Moreover, yeast one-hybrid screens showed that *PybZIPa* binds to the tandem G-box elements in the *PyUFGT* promoter and to the G-box elements in the *PyMYB* and *PyBBX* promoters as well as its own promoter (Fig. 6b). This regulatory mechanism identified in this study is different from what is reported in *Arabidopsis*, apple and rice^47–49^, in that *PybZIPa* alone is able to transcriptionally activate target genes. Taken together, the results indicate that *PybZIPa* regulates light-induced anthocyanin biosynthesis by regulating not only important transcription factor genes such as *PyMYB and PyBBX* but also structural genes (*PyUFGT*) and undergoes self-binding (*PybZIPa* activates its own promoter) to amplify the activation effect.

## Discussion

### Light-induced anthocyanin components can be divided into two categories

We identified 89, 94, and 71 differentially accumulated metabolites in B1/A1, B2/A2, and B3/A3, respectively. According to the abundance and composition of the differentially accumulated metabolites, intermediate product accumulation occurred at the early stage (B1/A1) of light-induced anthocyanin accumulation, after which an increase in anthocyanin biosynthesis by the structural genes and regulatory genes involved in anthocyanin biosynthesis occurred in the later stages (B2/A2 and B3/A3). The KEGG pathway analysis results for the differentially accumulated metabolites showed similar results; the TCA cycle, starch and sucrose metabolism, and the biosynthesis of phenylpropanoids for intermediate product accumulation were significantly enriched in B1/A1, while the phenylpropanoid and flavonoid metabolism pathways were significantly enriched during the extremely high anthocyanin biosynthesis stage (B3/A3) in particular (Fig. S3).

The GO analysis results showed that most of the DEGs were classified into three functional categories: the top 3 terms in each category were “metabolic process”, “cellular process” and “signal organism process” in the “Biological Process (BP)” category; “cell”, “cell part” and “membrane” in the “Cellular Component (CC)” category; and “binding”, “catalytic activity” and “transporter activity” in the “Molecular Function (MF)” category (Fig. S4). These GO terms indicate light-induced metabolic processes that occur during red pear formation. We have previously shown that the gene expression patterns of *PyCOP1*, *PyDET1*, *PyPIF* and *PyHY5* are responsive to light after bag removal in a light-sensitive pear variety. Additionally, the expression of the anthocyanin-related biosynthesis genes *PyDFR*, *PyANS*, and *PyUFGT* and the transcription factor genes *PyMYB10*, *PybHLH33*, and *PyWD40* show a positive correlation with light-induced anthocyanin accumulation^50^. The expression patterns and GO categories of the above genes were analyzed in the current study (Fig. S5). They were differentially expressed in the bagged and debagged groups (Fig. S5A), and the GO terms classified them as being related to “anthocyanin-containing compound metabolic process”, “pigment metabolic process”, “photomorphogenesis”, and the “response to light stimulus” (Fig. S5B).

### A photosensitive *PybZIP* gene plays an important role during light-induced anthocyanin biosynthesis

*HY5*, a typical bZIP transcription factor, is a photoresponsive gene that has been reported to regulate anthocyanin biosynthesis through certain transcription factors^49^. In *Arabidopsis*, *AtHY5* is directly activated by *AtBBX21*^51^. Similar findings in rice showed that anthocyanin biosynthesis is regulated by *OsBBX14* together with *OsHY5*^47^. The promotion of anthocyanin biosynthesis has also been found to occur through an increase in *MYB75/PAP1* transcriptional activity in *Arabidopsis*^49^. Similarly, *MdHY5* from apple accelerates anthocyanin accumulation by upregulating the expression of the *MdMYB10* transcription factor^48^. In this study, according to correlation analysis between the transcriptome and metabolome, another *PybZIP* transcription factor was shown to be highly correlated with Cy, which showed a strong response to light. In comparison to *HY5*, *PybZIPa* was more sensitive to light. The overexpression of *PybZIPa* promoted anthocyanin accumulation in pear, strawberry fruit, and tobacco leaves. Dual luciferase reporter and Y1H assays further confirmed that *PybZIPa* activated the transcription of the coexpressed genes *PyUFGT*, *PyMYB114*, *PyMYB10*, and *PyBBX22* by binding to G-box motifs in the promoters of these genes. In addition, *PybZIPa* activated its own promoter.

### Multiomics analysis reveals new links between transcription and metabolism

Along with the development and improvement of sequencing strategies and technology, a systems approach for different levels of omics analyses of the mechanisms underlying a physiological phenomenon has become possible. A large number of DEGs and annotated metabolic pathways can be obtained with transcriptome sequencing technology. However, there is not a simple connection between the transcriptome and phenotype. Metabolites are the final products of whole metabolic pathways; hence, the metabolome can serve as an important bridge between the genome and phenotype^52^. To gain an accurate understanding of biological networks, information from biological-omics data should be integrated. Multiomics analysis has established the foundation for new strategies for subsequent research. Correlation analysis between the transcriptome and metabolome reveals differentially accumulated metabolites that are related to phenotypic change and the DEGs that cause the changes in metabolites. This integrative analysis approach makes it easier to identify key regulatory metabolic pathways and reliable key regulatory genes^29,34,37^.

**Fig. 7:**
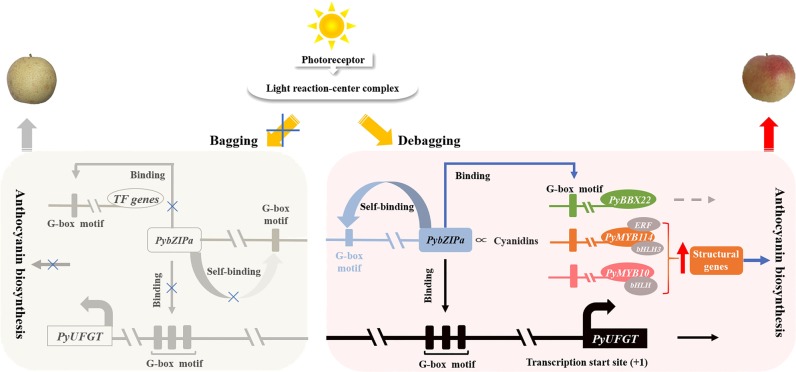
Light-induced anthocyanin biosynthesis regulation model in pear skin.

In this study, we used metabolomic and transcriptomic profiling to characterize the complex interplay between pear skin metabolomics and the corresponding transcriptomic data. We made full use of the correlations between different omics levels to filter redundant information to avoid making wild guesses based on big data and give full play to the advantages of multiomics. Using this method, we identified a photosensitive *PybZIPa* gene that promotes anthocyanin accumulation by binding to the promoter of *PyUFGT*, the anthocyanin biosynthesis-associated transcription factors *PyMYB10, PyMYB114*, and *PyBBX22*, and its own promoter. Based on this and previous studies, we propose a regulatory network for anthocyanin biosynthesis in pear (Fig. 7). Our results show the significance of integrated multiomics approaches for understanding plant physiological processes and provide a case study of the analysis of molecular mechanisms based on multiomics.

## Conclusion

We performed metabolomic (based on LC/MS) and transcriptomic analysis (based on high-throughput sequencing) of bagged and debagged pear fruit. Cyanidin and its derivatives were the main anthocyanin compounds involved in light-induced coloration. The integrated analysis of the transcriptome and metabolome showed that a novel photosensitive *PybZIPa* gene promoted light-induced anthocyanin accumulation by binding tandem G-box motifs in the *PyUFGT* promoter. In addition, *PybZIPa* regulated the anthocyanin biosynthesis-associated transcription factors *PyMYB114*, *PyMYB10*, and *PyBBX22* by binding to their G-box motifs. In addition, *PybZIPa* activated its own promoter. Our results advance the understanding of the anthocyanin biosynthesis regulation network and provide molecular genetic information for the quality improvement of skin color in fruit.

## Materials and methods

### Plant material and collection

Fruits of “Mantianhong” pear (a red Chinese sand pear variety) (*Pyrus pyrifolia* Nakai) were collected from the Anning experimental station of the Yunnan Academy of Agricultural Sciences. The fruits were bagged in double-layered paper bags (yellow-black) 35 DAFB. The bags were randomly removed at 10 days before commercial maturity, and the fruits were re-exposed to sunlight. The fruits were randomly sampled at 4 (B1), 8 (B2), and 10 (B3) days after bag removal, and fruits from which the bags were not removed were sampled at the same time (A1, A2, A3) as a control. For each sample, three biological replicates were performed. All the fruits were peeled and immediately placed in liquid nitrogen, and the pericarp was then stored at −80 °C for the subsequent experiment.

### Metabolite extraction

High-performance liquid chromatography-grade acetonitrile, methanol, and l-2-chlorophenylalanine were used for metabolite extraction. Samples of 50 mg from each stage were accurately weighed and placed into an Eppendorf tube (1.5 mL) (the internal standard was 20 μL of 0.3 mg/mL l-2-chlorophenylalanine), which was vortexed for 10 s. Then, 1 mL of methanol/water (7/3, vol/vol) solution was added to the tube. After being placed at −80 °C for 3 min, the samples were homogenized at 60 Hz for 2 min. Then, the mixture was subjected to ultrasonic treatment for 30 min (25–28 °C), placed at −20 °C for 20 min, and centrifuged for 10 min at 14,000 rpm in precooled centrifuges (4 °C). An 800 μL aliquot of the supernatant was used for LC-MS analysis.

### Metabolite profiling using LC/MS

An Agilent 1290 Infinity UHPLC coupled with an Agilent 6538 UHD and Accurate-Mass Q-TOF/MS system (Agilent Technologies) was used for metabolic profiling analysis in positive- and negative-ion modes. In positive- and negative-ion modes, metabolite separation was conducted in a Waters ACQUITY UPLC@HSS T3 column (2.1 × 100 mm, 2.1 μm), and the mobile phase consisted of A and B (A = water with 0.1% formic acid, B = acetonitrile with 0.1% formic acid). The following program was used for the linear elution gradient: 5% B for 2 min, then a linear increase to 95% B at 13 min, followed by holding for 2 min. Each run took 15 min. The column temperature was kept at 40 °C, and the flow rate remained at 0.4 mL/min, with a 3 μL injection volume. Mass spectrometry detection was carried out according to the following procedures: gas temperature: 350 °C; nitrogen flow: 11 L/min; capillary voltage: 4 kV and 3.5 kV for positive- and negative-ion modes respectively; nozzle voltage: 750 V; and mass scanning from 50 to 1,100 Da.

Quality control samples (QC samples) were obtained by mixing aliquots of all samples. The QC samples were injected regularly while the analytical program was being run to obtain a dataset to be used for repeatability assessment. Before pattern recognition, the LC-MS data files were converted into the available general format by Agilent MassHunter Qualitative analysis software. The converted data were further analyzed by XCMS for peak integration and correction, which produced a three-dimensional data matrix composed of the peak intensity, mass-to-charge ratio and retention time. The resulting data from the positive- and negative-ion modes were normalized and merged into a new matrix.

### PCA and identification of differentially accumulated metabolites

The converted data were submitted to the SIMCA-P software package, v14.0 (Umetrics, Umeå, Sweden), in which unsupervised principal component analysis (PCA) and (O)PLS-DA [(orthogonal) partial least-squares discriminant analysis] of data from 49 samples (seven samples × seven biological replicates) were performed to investigate the metabolic composition changes after bag removal. The metabolites with a VIP value (variable influence on projection value) > 1, a *p* value < 0.05 (two-tailed Student’s *t* test, normalized peak areas) and a fold change > 1.5 were considered differentially accumulated metabolites. The METLIN database (https://metlin.scripps.edu/) was used to identify differentially accumulated metabolites.

### RNA sequencing (RNA-seq) and data analysis

Total RNA from each sample (A1, A2, A3, B1, B2, B3) was isolated and then detected on an agarose gel (1%), and the concentration and RNA integrity number (RIN) values of the RNAs were also assessed. After enrichment and purification, 3 µg RNA of each sample was used for sequencing. The NEBNext® Ultra™ RNA Library Prep Kit for Illumina® (NEB, USA) was used for sequencing library construction. After mRNA enrichment and purification, the sequences were fragmented to short lengths. These fragments were used as templates for first- and second-strand cDNA synthesis. With the help of exonuclease/polymerase activities, the remaining overhangs were transformed into blunt ends. The NEBNext Adaptor (a hairpin loop structure included) was ligated to the 3’ ends of the DNA fragments for hybridization after adenylation. AMPure XP beads (Beckman Coulter, Beverly, USA) were used for segment size selection. Before PCR, the adaptor-ligated cDNA was treated at 37 °C (15 min) and then at 95 °C (5 min). Phusion High-Fidelity DNA polymerase was used for PCR. Then, the AMPure XP system was used for PCR product purification, and the Agilent Bioanalyzer 2100 system was used for library quality assessment.

### Transcript profiles and annotation

Differential expression analysis between the bagged and debagged groups (with two biological replicates per group) was implemented with the DESeq R package (1.18.0). DESeq determines differential expression by using a negative binomial distribution-based model^53^. For false discovery rate (FDR) control, the Benjamini and Hochberg approach was used for *P*-value adjustment. An adjusted *P*-value < 0.05 was used to define differentially expressed genes between the bagged and debagged groups.

The GOseq R package was used for the GO (Gene Ontology) enrichment of differentially expressed genes (DEGs), and the corrected *P*-value was used to define the significantly enriched terms (<0.05). The KEGG pathway enrichment analysis of the DEGs was accomplished with KOBAS software.

### Correlation analysis of the metabolome and transcriptome

Pearson correlation coefficients were calculated for the integration of the metabolome and transcriptome information. The mean metabolite content according to the metabolome data and the mean expression of each transcript according to the transcriptome data were used for coefficient calculation. The fold changes in each group (bagged and debagged groups in the corresponding period, B1/A1, B2/A2, and B3/A3) were calculated for both the metabolome and transcriptome data. Pearson correlation coefficients > 0.9 and *P*-values < 0.05 were considered to indicate a significant correlation, and the top 10 transcripts for each anthocyanin derivatives were identified and visualized using Cytoscape (version 3.4.0). The top 10 transcripts for the anthocyanin derivatives were subjected to KEGG pathway enrichment analysis.

### Plants and growth conditions

Six-leaved plants (with the smallest leaves over 1 cm long) of *Nicotiana tabacum* and *Nicotiana benthamiana* (grown in a greenhouse at 22 °C with 16 h of light a day) can be used for infection^46,54^. Diploid strawberry (*Fragaria vesca*) “Yellow Wonder” 5AF7 was grown in a glasshouse at 23 °C with 12 h of light per day, 2 or 3 weeks after anthesis (the same fruit growth stage examined by Yao et al.)^46^, and the fruits could be infected. Pear (*Pyrus bretchneideri* cv. Zaosu) fruits for infection were collected at 20 days before harvest.

### Subcellular localization of PybZIPa

The vector construction and experimental procedures for determining the subcellular localization of *PybZIPa* were followed Xue et al.^55^ with little modification. The recombinant plasmid *PybZIPa*-GFP was used for *Agrobacterium* transformation (*Agrobacterium tumefaciens* strain GV3101) via the freeze-thaw method. The leaves of *Nicotiana benthamiana* plants (~3–4-weeks-old) with uniform growth were infiltrated through their abaxial surfaces (*Agrobacterium* suspension concentration OD_600_ = 0.5 ~ 0.6; the empty GFP vector was used as a control), and then the infiltrated plants were cultured in a greenhouse At 72 h after infiltration, the infiltrated leaf tissues were collected and stained in a DAPI (fluorescent dye) working solution. After vacuum treatment, the green fluorescence and nuclear DNA signals were observed using a Leica TCs SP2 spectral confocal microscope (Leica Microsystems, Germany). The microscope settings (such as the laser intensity, pinhole, and photomultiplier gain) were kept consistent between different samples to obtain comparable images. ZEN 2012 (blue edition) (Carl Zeiss Microscopy GmbH, Germany) and Adobe Photoshop Version 14.0 (Adobe Systems, USA) were used for image processing.

### Overexpression of *PybZIPa* in the transient transformation system

The transient assays in *N. tabacum, F. vesca* and *P. bretchneideri* cv. Zaosu were performed as previously described^46^. The full-length coding sequence (CDs) of *PybZIPa* was amplified and inserted into the pSAK277 transient expression vector with the 35 S promoter, and the recombinant plasmid was then transformed into *Agrobacterium* strain GV3101. The *Agrobacterium* strain was infiltrated on the tobacco abaxial leaf surface, strawberry fruitlets and pear peels for phenotype investigation, while *Agrobacterium* cultures carrying the empty vector served as the negative control. To control variability, at least two leaves or fruitlets were infiltrated. The leaves or fruitlets were harvested and photographed approximately ten days after infiltration.

### Dual luciferase assay and yeast one-hybrid (Y1H) assay

To detect the transcriptional activation of coexpressed genes by *PybZIPa*, the promoter sequences of *PyCHI* and *PyANS*, *PyUFGT*, *PyMYB114*, *PyMYB10*, *PybZIPa*, and *PyBBX22* were inserted into the pGreen 0800-LUC vector (reporter vector). Vector construction for the transient expression and dual-luciferase assay was performed according to Hellens et al.^54^. G-box mutations were introduced into the *PyUFGT* promoter to verify the importance of the tandem G-box according to the instructions of the Mut Express® MultiS Fast Mutagenesis Kit V2 (Vazyme Biotech Co., Ltd, China). Then, the correct recombinant plasmids were transformed into *Agrobacterium* strain GV3101 (with pSoup). The GV3101 *Agrobacterium* strain bacteria carrying *PybZIPa* and each promoter recombinant plasmid were cotransformed to *N. benthamiana* leaves^54^. LUC (Firefly luciferase) and REN (Renilla luciferase) activity was assayed according to the instructions of the Dual-Luciferase® Reporter Assay System (Promega, Madison, USA).

The CDs of *PybZIPa* and the cloned promoter fragments of *PyUFGT* and *PyMYB114*, *PyMYB10*, *PybZIPa* and *PyBBX22* were inserted into the pGADT7 vector and the pABAi vector, respectively. The primers for promoter fragment amplification are listed in Table S1. Screening was performed to determine the optimal concentration of AbA (Aureobasidin A) to suppress the bait reporter strain. The yeast strain Y1H Gold (Clontech, http://www.clontech.com) containing the correct recombinant pABAi vectors was grown on SD/-Ura screening medium (with different AbA concentrations). Then, the interactions between *PybZIPa* and the promoter fragments were detected on SD/-Leu medium with the optimal AbA concentration. Empty pGADT7 with the corresponding recombinant pABAi was used as a negative control, and p53-AbAi was used as a positive control.

## Supplementary information

Supplementary Table

Supplementary Figure

## References

[1] Wei YZ (2011). Differential expression of anthocyanin biosynthetic genes in relation to anthocyanin accumulation in the pericarp of *Litchi chinensis* Sonn. PLoS ONE.

[2] Azuma A, Yakushiji H, Koshita Y, Kobayashi S (2012). Flavonoid biosynthesis-related genes in grape skin are differentially regulated by temperature and light conditions. Planta.

[3] Niu SS (2010). Coordinated regulation of anthocyanin biosynthesis in Chinese bayberry (*Myrica rubra*) fruit by a R2R3 MYB transcription factor. Planta.

[4] Uleberg E (2012). Effects of temperature and photoperiod on yield and chemical composition of northern and southern clones of bilberry (*Vaccinium myrtillus* L.). J. Agric. Food Chem..

[5] Wang Z, Gerstein M, Snyder M (2009). RNA-Seq: a revolutionary tool for transcriptomics. Nat. Rev. Genet..

[6] Kadomura-Ishikawa Y, Miyawaki K, Noji S, Takahashi A (2013). Phototropin 2 is involved in blue light-induced anthocyanin accumulation in *Fragaria* × *ananassa* fruits. J. Plant Res..

[7] Jia HJ, Araki A, Okamoto G (2005). Influence of fruit bagging on aroma volatiles and skin coloration of ‘Hakuho’ peach (*Prunus persica* Batsch). Postharvest Biol. Tec..

[8] Ravaglia D (2013). Transcriptional regulation of flavonoid biosynthesis in nectarine (*Prunus persica*) by a set of R2R3 MYB transcription factors. BMC Plant Biol..

[9] Bai S (2017). Transcriptome analysis of bagging-treated red Chinese sand pear peels reveals light-responsive pathway functions in anthocyanin accumulation. Sci. Rep..

[10] Feng S, Wang Y, Yang S, Xu Y, Chen X (2010). Anthocyanin biosynthesis in pears is regulated by a R2R3-MYB transcription factor *PyMYB10*. Planta.

[11] Sun Y (2014). Postharvest pigmentation in red Chinese sand pears (*Pyrus pyrifolia* Nakai) in response to optimum light and temperature. Postharvest Biol. Tec..

[12] Takos AM (2006). Light-induced expression of a *MYB* gene regulates anthocyanin biosynthesis in red apples. Plant Physiol..

[13] Takos MA, Robinson PS, Walker RA (2015). Transcriptional regulation of the flavonoid pathway in the skin of dark-grown ‘Cripps’ Red’ apples in response to sunlight. J. Hortic. Sci. Biotech..

[14] Feng F, Li M, Ma F, Cheng L (2013). Phenylpropanoid metabolites and expression of key genes involved in anthocyanin biosynthesis in the shaded peel of apple fruit in response to sun exposure. Plant Physiol. Biochem..

[15] Kang CY, Lian HL, Wang FF, Huang JR, Yang HQ (2009). Cryptochromes, phytochromes, and COP1 regulate light-controlled stomatal development in *Arabidopsis*. Plant Cell.

[16] Ma L (2002). Genomic evidence for COP1 as a repressor of light-regulated gene expression and development in Arabidopsis. Plant Cell.

[17] Wu D (2012). Structural basis of ultraviolet-B perception by UVR8. Nature.

[18] Lau OS, Deng XW (2012). The photomorphogenic repressors COP1 and DET1: 20 years later. Trends Plant Sci..

[19] Stracke R (2010). The Arabidopsis bZIP transcription factor HY5 regulates expression of the PFG1/MYB12 gene in response to light and ultraviolet-B radiation. Plant Cell Environ..

[20] An, J. P. et al. *MdBBX22* regulates UV-B-induced anthocyanin biosynthesis through regulating the function of MdHY5 and is targeted by MdBT2 for 26S proteasome-mediated degradation. *Plant Biotechnol. J.* **17**, 2231–2233 (2019). 10.1111/pbi.13196 PMC6835122 31222855

[21] Yadav A (2019). The B-Box-containing microprotein miP1a/BBX31 regulates photomorphogenesis and UV-B protection. Plant Physiol..

[22] Heng Y (2019). B-Box containing proteins BBX30 and BBX31, acting downstream of HY5, negatively regulate photomorphogenesis in *Arabidopsis*. Plant Physiol..

[23] Bai SL (2014). An apple B-box protein, MdCOL11, is involved in UV-B- and temperature-induced anthocyanin biosynthesis. Planta.

[24] Casati P, Campi M, Morrow DJ, Fernandes J, Walbot V (2011). Transcriptomic, proteomic and metabolomic analysis of maize responses to UV-B: comparison of greenhouse and field growth conditions. Plant Signal Behav..

[25] Stushnoff C (2010). Flavonoid profiling and transcriptome analysis reveals new gene-metabolite correlations in tubers of *Solanum tuberosum* L. J. Exp. Bot..

[26] Kong SG, Okajima K (2016). Diverse photoreceptors and light responses in plants. J. Plant Res..

[27] Persson S, Wei H, Milne J, Page GP, Somerville CR (2005). Identification of genes required for cellulose synthesis by regression analysis of public microarray data sets. Proc. Natl Acad. Sci. USA.

[28] Sekhon RS (2012). Transcriptional and metabolic analysis of senescence induced by preventing pollination in maize. Plant Physiol..

[29] Ibáñez AM (2014). Transcriptome and metabolome analysis of citrus fruit to elucidate puffing disorder. Plant Sci..

[30] Caldana C (2011). High-density kinetic analysis of the metabolomic and transcriptomic response of Arabidopsis to eight environmental conditions. Plant J..

[31] Cho K (2008). Integrated transcriptomics, proteomics, and metabolomics analyses to survey ozone responses in the leaves of rice seedling. J. Proteome Res..

[32] Tan FQ (2015). Comparative metabolic and transcriptional analysis of a doubled diploid and its diploid citrus rootstock (C. junos cv. Ziyang xiangcheng) suggests its potential value for stress resistance improvement. BMC Plant Biol..

[33] Wang D, Amornsiripanitch N, Dong X (2006). A genomic approach to identify regulatory nodes in the transcriptional network of systemic acquired resistance in plants. PLoS Pathog..

[34] Cho K (2016). Network analysis of the metabolome and transcriptome reveals novel regulation of potato pigmentation. J. Exp. Bot..

[35] Hirai MY (2007). Omics-based identification of Arabidopsis Myb transcription factors regulating aliphatic glucosinolate biosynthesis. Proc. Natl Acad. Sci. USA.

[36] Gutierrez RA (2008). Systems approach identifies an organic nitrogen-responsive gene network that is regulated by the master clock control gene CCA1. Proc. Natl Acad. Sci. USA.

[37] Cohen H, Amir R (2017). Dose-dependent effects of higher methionine levels on the transcriptome and metabolome of transgenic Arabidopsis seeds. Plant Cell Rep..

[38] Hirai MY (2004). Integration of transcriptomics and metabolomics for understanding of global responses to nutritional stresses in *Arabidopsis thaliana*. Proc. Natl Acad. Sci. U. S. A..

[39] Lu XP (2017). Transcriptome and metabolome analyses provide insights into the occurrence of peel roughing disorder on Satsuma Mandarin (*Citrus unshiu* Marc.) fruit. Front. Plant Sci..

[40] Agudelo-Romero P (2015). Transcriptome and metabolome reprogramming in *Vitis vinifera* cv. Trincadeira berries upon infection with *Botrytis cinerea*. J. Exp. Bot..

[41] Fortes AM (2011). Transcript and metabolite analysis in Trincadeira cultivar reveals novel information regarding the dynamics of grape ripening. BMC Plant Biol..

[42] Li Y (2018). Combined analysis of the fruit metabolome and transcriptome reveals candidate genes involved in flavonoid biosynthesis in Actinidia arguta. Int. J. Mol. Sci..

[43] Yun Z (2016). Comparative transcriptome and metabolome provides new insights into the regulatory mechanisms of accelerated senescence in litchi fruit after cold storage. Sci. Rep..

[44] Wang Z, Cui Y, Vainstein A, Chen S, Ma H (2017). Regulation of Fig (*Ficus carica* L.) fruit color: metabolomic and transcriptomic analyses of the flavonoid biosynthetic pathway. Front. Plant Sci..

[45] Zhang H (2011). Genome-wide mapping of the HY5-mediated gene networks in Arabidopsis that involve both transcriptional and post-transcriptional regulation. Plant J..

[46] Yao G (2017). Map-based cloning of the pear gene MYB114 identifies an interaction with other transcription factors to coordinately regulate fruit anthocyanin biosynthesis. Plant J..

[47] Kim DH (2018). A Rice B-Box protein, OsBBX14, finely regulates anthocyanin biosynthesis in rice. Int. J. Mol. Sci..

[48] An JP (2017). The bZIP transcription factor MdHY5 regulates anthocyanin accumulation and nitrate assimilation in apple. Hortic. Res..

[49] Shin, D. H. et al. HY5 regulates anthocyanin biosynthesis by inducing the transcriptional activation of the MYB75/PAP1 transcription factor in. *Arabidopsis. FEBS Lett.* **587**, 1543–1547 (2013). 10.1016/j.febslet.2013.03.037 23583450

[50] Zhu YF (2018). Different light-response patterns of coloration and related gene expression in red pears (*Pyrus* L.). Sci. Hortic..

[51] Xu D (2016). BBX21, an Arabidopsis B-box protein, directly activates HY5 and is targeted by COP1 for 26S proteasome-mediated degradation. Proc. Natl Acad. Sci. USA.

[52] Nobeli I, Thornton JM (2006). A bioinformatician’s view of the metabolome. Bioessays.

[53] Anders S, Huber W (2010). Differential expression analysis for sequence count data. Genome Biol..

[54] Hellens RP (2005). Transient expression vectors for functional genomics, quantification of promoter activity and RNA silencing in plants. Plant Methods.

[55] Xue C (2019). *PbrMYB169* positively regulates lignification of stone cells in pear fruit. J. Exp. Bot..

