# Supplementary material for: The involvement of PybZIPa in light-induced anthocyanin accumulation via the activation of PyUFGT through binding to tandem G-boxes in its promoter
Source: Hortic Res. 2019 Dec 1;6:134. doi: 10.1038/s41438-019-0217-4 (PMC6885052; doi:10.1038/s41438-019-0217-4)
Supplement: Supplementary file 1 — Supplementary Table [file 41438_2019_217_MOESM1_ESM.pdf]

Table S1. Primers used in this study.

Primer used	*Name*	Forward primer (5' to 3')	Reverse primer (5' to 3')
For qPCR	*Tub(reference gene)*	TGGGCTTTGCTCCTCTTAC	CCTTCGTGCTCATCTTACC
	PybZIPa	CTACAATCGCAAATGCCACC	CACCCTGTTCCTTAGCAATCTC
	PyCHI	AAGGATTCCGTCATCACAT	GTCCCACCCAAATACCAT
	PyF3'H	CCACATCTAGCCGTTGA	CCCACAGTTTAGGGTCTC
	PyANS	AAGTATGCCAATGACCAGG	GCTCTTCAAGTCCACCAAC
	PyUFGT	CCCGTTGTCGGTGTTGTT	ATCCGCATAGTCGCTTGG
	PyMYB114	CAAAAGCCACATCCGTCATAA	AGGTCTTCCCCTAACCCTAAACT
	PyMYB10	CACAAACGTCGTCGTCAACAA	CCGACAGTCGATCATCAAACC
	PyBBX22	AAGGTTCACAAAGCGAATAAGC	GAAGACGCCCCAACACAA
For overexpression vector	*pSAK277 (sequencing primer)*	CATCGAAAGGACAGTAGAAAAGG	CATTAGAATGAACCGAAACCG
	pSAK-PybZIPa	actagtggatccaaagaattcATGTCAGTCCCAATCAGAGC	tcattaaagcaggactctagaCTTAACTGATCCTTGC
For subcellular localization	*GFP (sequencing primer)*	TCCACTGACGTAAGGGATG	CGTCGTCCTTGAAGAAGATG
	PybZIPa-GFP	gagaacacgggggactctagaATGTCAGTCCCAATCAGAGCAGG	gcccttgctcaccatggatccCTTAACTGATCCTTGCTTTTGCTCG
For dual luciferase assay	*0800-proCHI*	gtcgacggtatcgataagcttCAGCACGGAGGAGTTTCT	agaactagtggatcccccgggCATCTTTGCACCTGTTCG
	0800-proANS	gtcgacggtatcgataagcttCAATAGGCTACCAATAACG	agaactagtggatcccccgggACCCACCAGACTTTCATC
	0800-proUFGT	gtcgacggtatcgataagcttCCTTGTGGGTAATGGTTT	agaactagtggatcccccgggAAATAACAGTGGGACCTA
	0800-proMYB114	gtcgacggtatcgataagcttTCATTAAAAATTGCTAATTACATTCTTAATATT	agaactagtggatcccccgggTATTCCCAGCAAATTACTATCCTCC
	0800-proMYB10	gtcgacggtatcgataagcttAGTTAGTAGAATTATCTAAATATCTCTAACATGATG	agaactagtggatcccccgggCTCGTATCTGCTAGCAGCTAAGCT
	0800-proBBX22	gtcgacggtatcgataagcttGTCGAAAATTGATAGGTGTGTGTGA	agaactagtggatcccccgggCGGGGAGGATCCTGTTGACA
	0800-probZIPa	gtcgacggtatcgataagcttTCTTTTACATTGAAAAGGCCTTTCC	agaactagtggatcccccgggGGCTGGCTTATGAGCTCTCTCTC
For yeast one-hybrid assay	*pAbAi-vector (sequencing primer)*	TTCAAGGAAACCGAAATCA	TATGCTACAAAGGACCTAAT
	pGADT7-vector (sequencing primer)	TAATACGACTCACTATAGGGC	AGATGGTGCACGATGCACAG
	pGAD-PybZIPa	ggccagtgaattccacccgggATGTCAGTCCCAATCAGAGC	acgattcatctgcagctcgagCTTAACTGATCCTTGC
	pABAi-proUFGT-G-box	cttgaattcgagctcggtaccGTGGTAATCAAGGAACCACG	atacagagcacatgcctcgagTGCGGTTGAGATTTCCAGTT
	pABAi-proMYB114-G-box	cttgaattcgagctcggtaccATCTTGCCTAGCAAGTATTATGGTCG	atacagagcacatgcctcgagGGGGGAAATATTTAGAGAAAGAGAAA
	pABAi-proMYB10-G-box	cttgaattcgagctcggtaccCTGCCTTCACATTCATAGAATGTGC	atacagagcacatgcctcgagGGGGAAATTTTTAGAGAAAGAGAAAC
	pABAi-proBBX22-G-box	cttgaattcgagctcggtaccGTCGAAAATTGATAGGTGTGTGTGA	atacagagcacatgcctcgagCGGGGAGGATCCTGTTGACA
	pABAi-probZIPa-G-box	cttgaattcgagctcggtaccACTTGCAATATGTACATATTTTTTACTTAGG	atacagagcacatgcctcgagAACTAGTGGATCCCCCGGG

Note: The lowercase letters represent the restriction sites and homologous recombination arm for different vector constructions.

Table S2. Interaction value between metabolites and genes

ID-1	ID-2	Pearson	*p-value*
Kaempferol 4'-rhamnoside	Pbr020478.1	0.999998848	6.04E-05
Kaempferol 4'-rhamnoside	Pbr040222.1	0.999994193	0.000135603
Kaempferol 4'-rhamnoside	Pbr014872.1	0.999978767	0.000259285
Kaempferol 4'-rhamnoside	Pbr030373.1	0.999976205	0.000274485
Kaempferol 4'-rhamnoside	Pbr035932.1	0.999963003	0.000342261
Kaempferol 4'-rhamnoside	Pbr015347.1	0.999959088	0.000359918
Kaempferol 4'-rhamnoside	Pbr023848.1	0.999945847	0.000414086
Kaempferol 4'-rhamnoside	Pbr003806.1	0.999919611	0.000504518
Kaempferol 4'-rhamnoside	Pbr014148.1	0.999900839	0.000560337
Kaempferol 4'-rhamnoside	Pbr003780.1	0.999842685	0.000705774
6-Hydroxykaempferol 3-methyl ether 6-glucoside	Pbr007194.1	0.999980399	0.000249126
6-Hydroxykaempferol 3-methyl ether 6-glucoside	Pbr007004.1	0.999972155	0.000296928
6-Hydroxykaempferol 3-methyl ether 6-glucoside	Pbr025647.1	0.999969673	0.000309879
6-Hydroxykaempferol 3-methyl ether 6-glucoside	Novel00072	0.999961585	0.000348758
6-Hydroxykaempferol 3-methyl ether 6-glucoside	Pbr035967.1	0.99992632	0.000483005
6-Hydroxykaempferol 3-methyl ether 6-glucoside	Pbr032910.1	0.999882153	0.000610856
6-Hydroxykaempferol 3-methyl ether 6-glucoside	Pbr009585.1	0.999834762	0.000723329
6-Hydroxykaempferol 3-methyl ether 6-glucoside	Pbr019926.1	0.999822341	0.000750023
6-Hydroxykaempferol 3-methyl ether 6-glucoside	Pbr021355.1	0.999812258	0.000771015
6-Hydroxykaempferol 3-methyl ether 6-glucoside	Pbr033501.1	0.982752663	0.007400522
Cyanidin 3-(6-malylglucoside)	Pbr005128.2	0.999981177	0.000244129
Cyanidin 3-(6-malylglucoside)	Pbr008923.1	0.999845048	0.000700454
Cyanidin 3-(6-malylglucoside)	Pbr038552.1	0.999731606	0.000921873
Cyanidin 3-(6-malylglucoside)	Pbr015483.1	0.999720292	0.000941105
Cyanidin 3-(6-malylglucoside)	Pbr013884.1	0.999633493	0.001077282
Cyanidin 3-(6-malylglucoside)	Pbr040794.1	0.999492528	0.00126765
Cyanidin 3-(6-malylglucoside)	Pbr039999.1	0.999475235	0.00128907
Cyanidin 3-(6-malylglucoside)	Novel00370	0.948484846	0.012826994
Cyanidin 3-(6-malylglucoside)	Pbr039972.1	0.934770085	0.014450672
Cyanidin 3-(6-malylglucoside)	Pbr039986.1	0.903587387	0.01761549
dimethoxyflavanone	Pbr019475.1	0.999996312	0.000108064
dimethoxyflavanone	Pbr022226.1	0.999994444	0.00013264
dimethoxyflavanone	Pbr030951.1	0.999972591	0.000294595
dimethoxyflavanone	Pbr019219.1	0.999966008	0.000328069
dimethoxyflavanone	Pbr029321.1	0.999898079	0.000568082
dimethoxyflavanone	Pbr017718.1	0.999866379	0.000650455
dimethoxyflavanone	Pbr029872.1	0.99984951	0.000690294
dimethoxyflavanone	Pbr025648.1	0.999809836	0.000775971
dimethoxyflavanone	Pbr031347.1	0.999773333	0.000847183
dimethoxyflavanone	Pbr023137.1	0.999769353	0.000854589
hexamethoxyflavone	Pbr004370.1	0.999994175	0.000135813
hexamethoxyflavone	Pbr013303.1	0.999904549	0.000549755
hexamethoxyflavone	Pbr035481.1	0.999724345	0.00093426
hexamethoxyflavone	Pbr013256.1	0.999583494	0.00114842
hexamethoxyflavone	Pbr035362.1	0.999172121	0.001619156
hexamethoxyflavone	Pbr019804.1	0.999144212	0.001646225
hexamethoxyflavone	Pbr029778.1	0.999082506	0.001704551
hexamethoxyflavone	Pbr009096.1	0.998873619	0.001888681
hexamethoxyflavone	Pbr028053.1	0.998659769	0.002060219
hexamethoxyflavone	Pbr042065.1	0.998414798	0.002240654
Kaempferol 3-glucuronide	Pbr023304.1	0.999987714	0.000197231
Kaempferol 3-glucuronide	Pbr035573.1	0.999963067	0.000341968
Kaempferol 3-glucuronide	Pbr032572.1	0.999961943	0.00034713

Kaempferol 3-glucuronide	Pbr010756.1	0.999946231	0.000412613
Kaempferol 3-glucuronide	Pbr039023.1	0.999887389	0.000597132
Kaempferol 3-glucuronide	Pbr017028.1	0.999866412	0.000650375
Kaempferol 3-glucuronide	Pbr030955.1	0.999862457	0.000659933
Kaempferol 3-glucuronide	Pbr015168.1	0.99984823	0.000693225
Kaempferol 3-glucuronide	Pbr003752.1	0.978390428	0.008286734
Kaempferol 3-glucuronide	Pbr033501.1	0.997832495	0.002620195
Quercetin 3-O-glucoside	Pbr000582.1	0.949467001	0.01270307
Quercetin 3-O-glucoside	Pbr003752.1	0.913168579	0.016703496
Quercetin 3-O-glucoside	Pbr021057.1	0.992808611	0.004774656
Quercetin 3-O-glucoside	Pbr010673.1	0.984257157	0.007069491
Quercetin 3-O-glucoside	Pbr001279.1	0.966166812	0.010379555
Quercetin 3-O-glucoside	Pbr031407.1	0.936556729	0.014249223
Quercetin 3-O-glucoside	Pbr030848.1	0.998861317	0.001898969
Quercetin 3-O-glucoside	Pbr039986.1	0.941851634	0.013635503
Quercetin 3-O-glucoside	Pbr039972.1	0.912174043	0.01680032
Quercetin 3-O-glucoside	Pbr002375.1	0.971967763	0.0094433
6-Methoxykaempferol 3-(6-acetylglucoside)	Pbr003128.1	0.999968257	0.000317032
6-Methoxykaempferol 3-(6-acetylglucoside)	Pbr031070.1	0.999959938	0.000356159
6-Methoxykaempferol 3-(6-acetylglucoside)	Pbr004201.3	0.999784601	0.000825857
6-Methoxykaempferol 3-(6-acetylglucoside)	Pbr041897.1	0.999769743	0.000853866
6-Methoxykaempferol 3-(6-acetylglucoside)	Pbr033827.1	0.999748813	0.000891831
6-Methoxykaempferol 3-(6-acetylglucoside)	Pbr004235.1	0.999660317	0.001037109
6-Methoxykaempferol 3-(6-acetylglucoside)	Pbr020567.1	0.999650623	0.001051805
6-Methoxykaempferol 3-(6-acetylglucoside)	Pbr017929.1	0.999327093	0.001459746
6-Methoxykaempferol 3-(6-acetylglucoside)	Pbr012103.1	0.999172541	0.001618745
6-Methoxykaempferol 3-(6-acetylglucoside)	Pbr038278.1	0.999030781	0.001751948
6-Hydroxydelphinidin 3-(6-malonylglucoside)	Pbr038278.1	0.999950326	0.00039659
6-Hydroxydelphinidin 3-(6-malonylglucoside)	Pbr042779.1	0.999941977	0.000428625
6-Hydroxydelphinidin 3-(6-malonylglucoside)	Pbr034311.1	0.999898057	0.000568143
6-Hydroxydelphinidin 3-(6-malonylglucoside)	Pbr017929.1	0.999850164	0.000688792
6-Hydroxydelphinidin 3-(6-malonylglucoside)	Pbr001290.1	0.999761557	0.000868912
6-Hydroxydelphinidin 3-(6-malonylglucoside)	Pbr042332.1	0.999715458	0.000949201
6-Hydroxydelphinidin 3-(6-malonylglucoside)	Pbr035938.1	0.999658021	0.001040608
6-Hydroxydelphinidin 3-(6-malonylglucoside)	Pbr004235.1	0.999609892	0.001111429
6-Hydroxydelphinidin 3-(6-malonylglucoside)	Pbr031120.1	0.999600034	0.001125385
6-Hydroxydelphinidin 3-(6-malonylglucoside)	Pbr038777.3	0.999587938	0.001142276
Apigenin 7-(6-malonylglucoside)	Pbr006275.1	0.999998293	7.35E-05
Apigenin 7-(6-malonylglucoside)	Pbr007711.1	0.999909802	0.000534413
Apigenin 7-(6-malonylglucoside)	Pbr037476.1	0.999907631	0.000540805
Apigenin 7-(6-malonylglucoside)	Pbr015252.1	0.999879256	0.000618319
Apigenin 7-(6-malonylglucoside)	Pbr019167.1	0.999835324	0.000722098
Apigenin 7-(6-malonylglucoside)	Pbr013387.1	0.999822935	0.000748769
Apigenin 7-(6-malonylglucoside)	Pbr031010.1	0.999808473	0.000778748
Apigenin 7-(6-malonylglucoside)	Pbr012355.1	0.999791511	0.000812502
Apigenin 7-(6-malonylglucoside)	Pbr037470.1	0.999634578	0.001075687
Apigenin 7-(6-malonylglucoside)	Pbr041824.1	0.999297333	0.001491679
prenylflavanone	Pbr030184.1	0.999997853	8.25E-05
prenylflavanone	Pbr026905.1	0.999994442	0.000132664
prenylflavanone	Pbr038288.1	0.999971811	0.000298753
prenylflavanone	Pbr029842.1	0.99996418	0.000336775
prenylflavanone	Pbr039230.1	0.99994411	0.000420672
prenylflavanone	Pbr022800.1	0.999915488	0.000517295
prenylflavanone	Pbr019343.1	0.999914042	0.000521701
prenylflavanone	Pbr038211.1	0.999908103	0.000539421

prenylflavanone	Pbr013360.1	0.999829599	0.000734543
prenylflavanone	Pbr033727.1	0.99051805	0.005483625
Quercetin 3-xyloside-7-glucoside	Pbr041904.1	0.999999651	3.33E-05
Quercetin 3-xyloside-7-glucoside	Pbr040178.1	0.999999118	5.28E-05
Quercetin 3-xyloside-7-glucoside	Pbr020773.1	0.999923193	0.000493148
Quercetin 3-xyloside-7-glucoside	Pbr034768.1	0.999917428	0.000511324
Quercetin 3-xyloside-7-glucoside	Pbr024972.1	0.999786824	0.000821584
Quercetin 3-xyloside-7-glucoside	Pbr018708.1	0.999782959	0.000828998
Quercetin 3-xyloside-7-glucoside	Pbr038099.1	0.999688184	0.000993655
Quercetin 3-xyloside-7-glucoside	Pbr036221.1	0.999551111	0.001192232
Quercetin 3-xyloside-7-glucoside	Pbr000414.1	0.999483603	0.00127875
Quercetin 3-xyloside-7-glucoside	Pbr033501.1	0.962135109	0.010984328
Quercetin 3-(2-glucosylrhamnoside)	Pbr000498.1	0.99999749	8.92E-05
Quercetin 3-(2-glucosylrhamnoside)	Pbr028471.1	0.999994227	0.000135204
Quercetin 3-(2-glucosylrhamnoside)	Pbr042698.1	0.999993525	0.000143182
Quercetin 3-(2-glucosylrhamnoside)	Pbr022330.1	0.999992276	0.000156383
Quercetin 3-(2-glucosylrhamnoside)	Pbr003581.1	0.999984798	0.000219394
Quercetin 3-(2-glucosylrhamnoside)	Pbr021355.1	0.999949296	0.000400682
Quercetin 3-(2-glucosylrhamnoside)	Pbr019926.1	0.999943844	0.000421673
Quercetin 3-(2-glucosylrhamnoside)	Pbr009585.1	0.999936509	0.000448368
Quercetin 3-(2-glucosylrhamnoside)	Pbr003752.1	0.908686872	0.017135755
Quercetin 3-(2-glucosylrhamnoside)	Pbr033501.1	0.987771452	0.006228825
Petunin	Pbr004849.1	0.999999934	1.44E-05
Petunin	Pbr020363.1	0.999999697	3.10E-05
Petunin	Pbr040057.1	0.999997347	9.16E-05
Petunin	Pbr023911.1	0.999993665	0.000141629
Petunin	Pbr041968.1	0.99997611	0.000275033
Petunin	Pbr031922.1	0.999976003	0.000275646
Petunin	Pbr016970.1	0.999968231	0.000317161
Petunin	Pbr016506.1	0.999964724	0.000334207
Petunin	Novel00448	0.999961311	0.000350003
Petunin	Pbr000218.1	0.991210505	0.005279292
Quercetin-glucoside	Pbr017672.1	0.999992371	0.000155425
Quercetin-glucoside	Pbr014325.1	0.999989248	0.000184512
Quercetin-glucoside	Pbr024458.1	0.999914465	0.000520414
Quercetin-glucoside	Pbr022187.1	0.999830318	0.000732991
Quercetin-glucoside	Pbr000452.1	0.999819315	0.000756384
Quercetin-glucoside	Pbr009881.1	0.999815262	0.000764821
Quercetin-glucoside	Pbr005912.1	0.999792364	0.000810838
Quercetin-glucoside	Pbr002886.1	0.999774284	0.000845405
Quercetin-glucoside	Pbr029551.1	0.999770194	0.000853029
Quercetin-glucoside	Pbr031958.1	0.991223616	0.005275348
Cyanidin-malonylglucoside	Pbr023911.1	0.999999601	3.55E-05
Cyanidin-malonylglucoside	Pbr020363.1	0.999998218	7.51E-05
Cyanidin-malonylglucoside	Pbr004849.1	0.999995413	0.000120519
Cyanidin-malonylglucoside	Pbr040057.1	0.999987649	0.000197753
Cyanidin-malonylglucoside	Pbr016506.1	0.999983567	0.000228102
Cyanidin-malonylglucoside	Pbr020016.1	0.999963146	0.000341602
Cyanidin-malonylglucoside	Pbr041968.1	0.999954121	0.000381139
Cyanidin-malonylglucoside	Pbr031922.1	0.999953973	0.000381752
Cyanidin-malonylglucoside	Pbr016970.1	0.999943419	0.000423267
Cyanidin-malonylglucoside	Pbr000218.1	0.990854189	0.005385398

**Table S3.** Comparative analysis of protein property				
Gene ID		Pbr002622.1	Pbr027818.1	*PybZIPa*
Formula		$C_{759}H_{1283}N_{253}O_{268}S_3$	$C_{735}H_{1239}N_{245}O_{261}S_3$	$C_{1033}H_{1680}N_{318}O_{353}S_5$
Mw		18337.25	17780.59	24362.92
Theoretical pI		9.34	9	5.58
Instability index		55.72	53.2	58.95
Aliphatic index		70.59	68.54	68.85
GRAVY		-0.931	-0.984	-1.036
Signal peptide		No	No	No
Number of phosphorylation sites		24	23	26
Protein secondary structure prediction	Alpha helix (%)	55.03	48.17	37.16
	Random coil (%)	42.6	45.12	47.25
	Extended strand (%)	1.18	2.44	14.22
	Beta turn (%)	1.18	4.27	1.38
Transmembrane prediction		No	No	No

Instability index > 40, the protein is probably unstable; GRAVY < 0 , the protein is probably hydrophilous.

Note: pI, isoelectric point; Mw, molecular weight; GRAVY, Grand average of hydropathicity. Protein property analysis was used ExPASy ProtParam tool (https://web.expasy.org/protparam/), ProtScale (http://web.expasy.org/protscale/), SignalP 4.1 server (http://www.cbs.dtu.dk/services/ SignalP/), NetPhos 2.0 Server (http://www.cbs.dtu.dk/services/NetPhos/) and Scratch Protein Predicter-DIpro (http://scratch.proteomics.ics.uci.edu/). Protein secondary structure prediction was used PBIL LYON-GERLAND (https://npsa-prabi.ibcp.fr/cgi-bin/npsa_automat.pl?page=/NPSA/npsa_hnn. html), transmembrane prediction was used TMHMM server v. 2.0 (http://genome.cbs.dtu.dk/services/TMHMM/).

Table S4. Correlation analysis of expression patterns

ID1	ID2	pearsonr
PybZIPa	*PyMYB114*	0.858792204
PybZIPa	*PyMYB10*	0.799350935
PybZIPa	*PyBBX22*	0.769090018
PybZIPa	*PyCHI*	0.778885835
PybZIPa	*PyF3'H*	0.827941627
PybZIPa	*PyANS*	0.885189748
PybZIPa	*PyUFGT*	0.927060622
