# Supplementary figures and images for: The involvement of PybZIPa in light-induced anthocyanin accumulation via the activation of PyUFGT through binding to tandem G-boxes in its promoter
Source: Hortic Res. 2019 Dec 1;6:134. doi: 10.1038/s41438-019-0217-4 (PMC6885052; doi:10.1038/s41438-019-0217-4)

Fig. S1

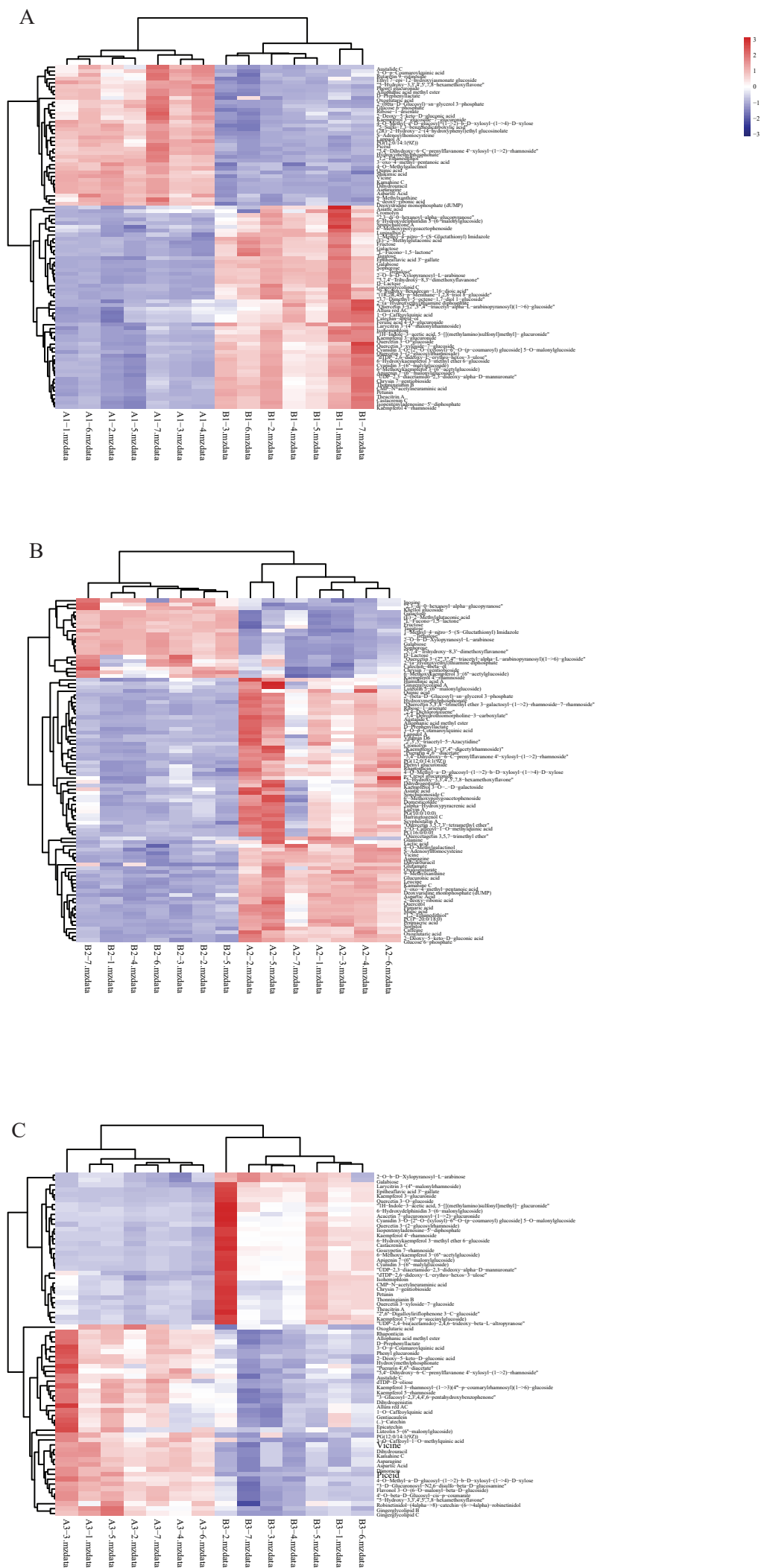

Fig. S2

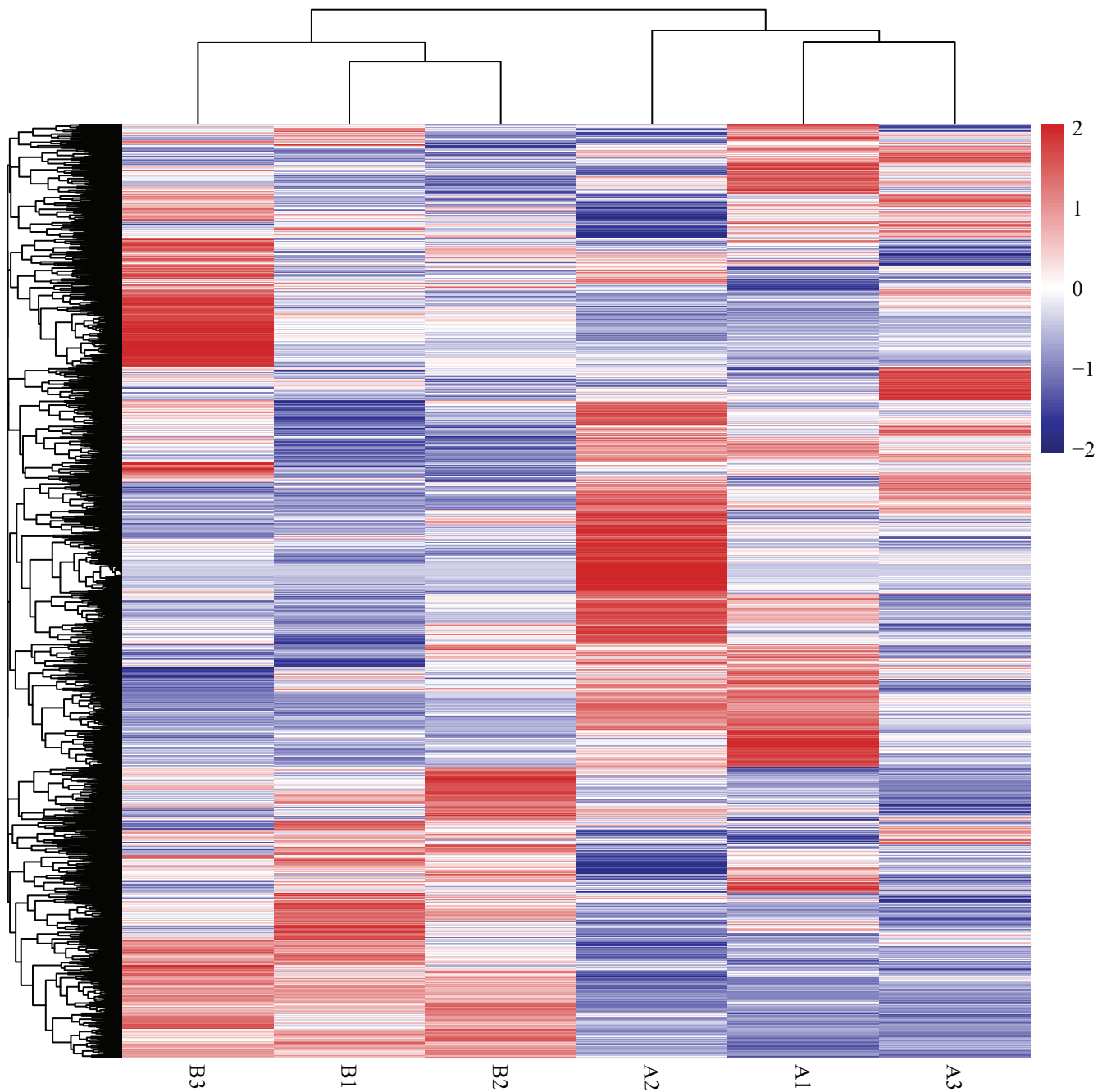

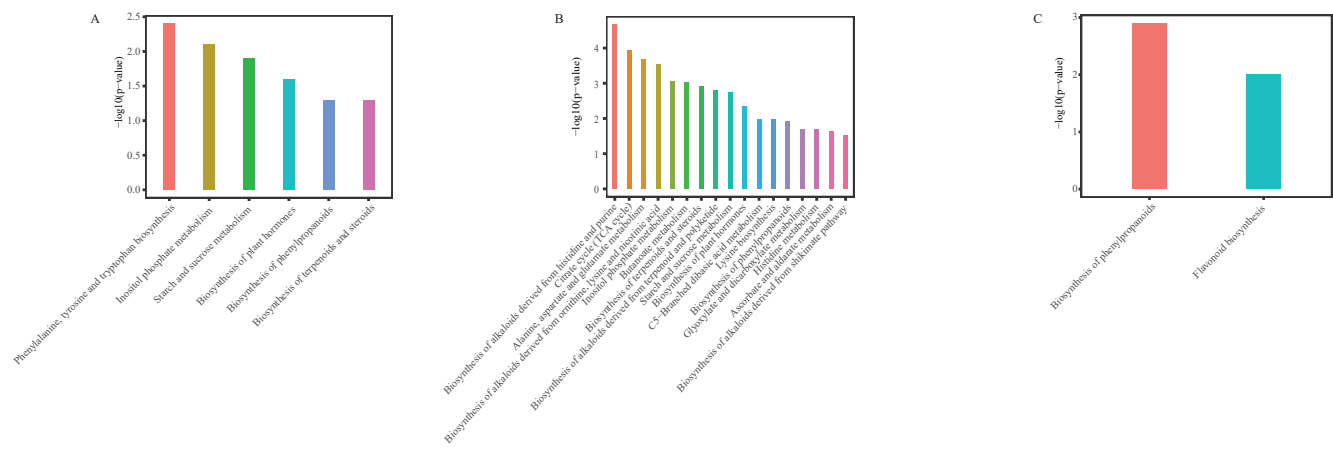

Fig. S4

Go enrichment analysis on the common DEGs

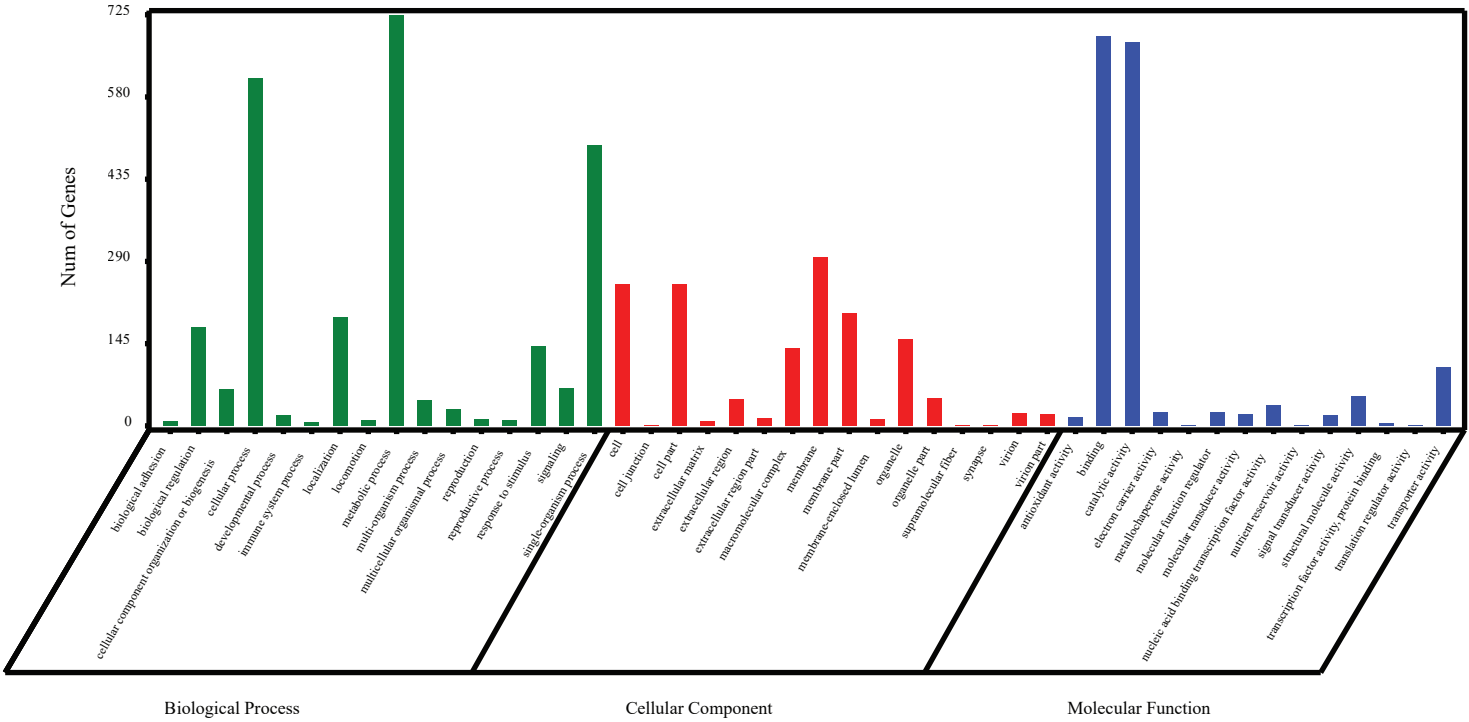

A

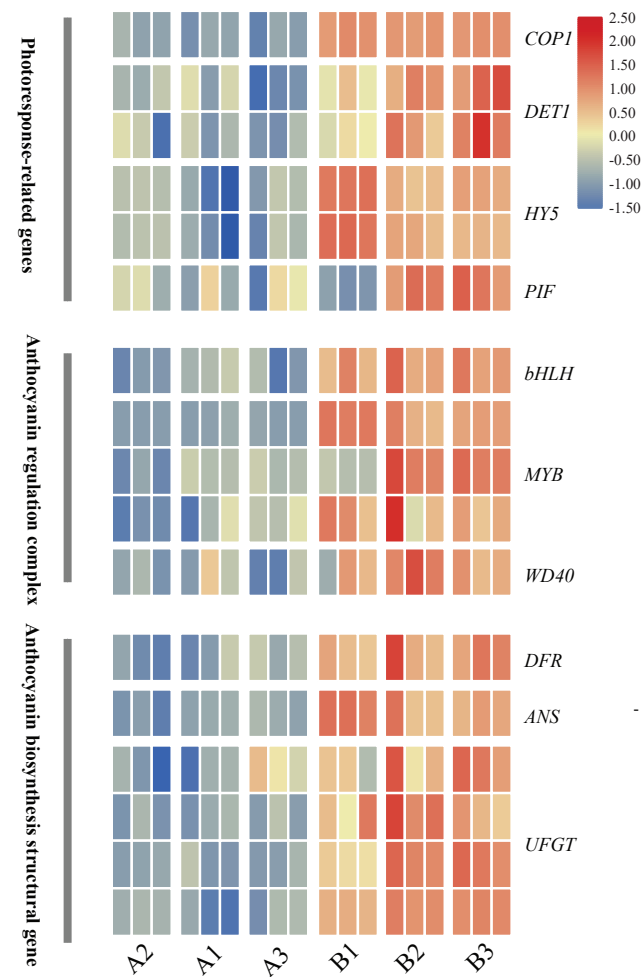

B

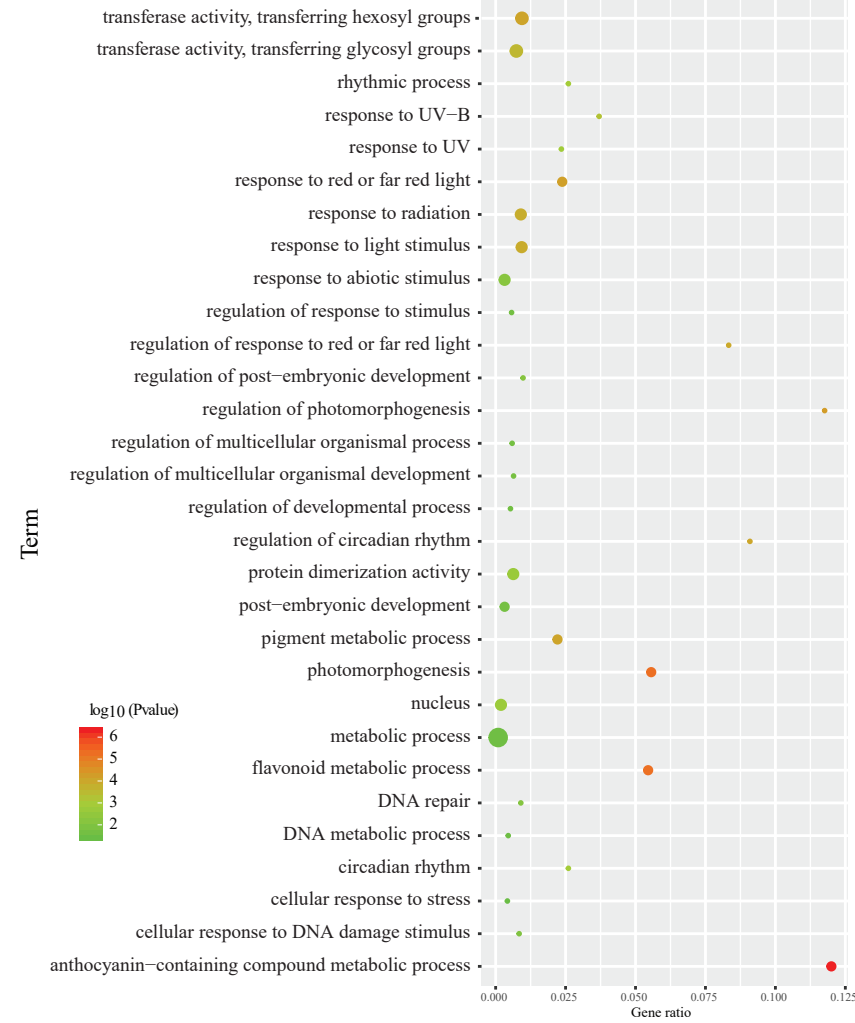

Fig.S5

Supplement: Supplementary file 2 — Supplementary Figure [file 41438_2019_217_MOESM2_ESM.pdf]
